# Iron Regulatory Mechanisms in *Saccharomyces cerevisiae*

**DOI:** 10.3389/fmicb.2020.582830

**Published:** 2020-09-09

**Authors:** Lucía Ramos-Alonso, Antonia María Romero, María Teresa Martínez-Pastor, Sergi Puig

**Affiliations:** ^1^Departamento de Biotecnología, Instituto de Agroquímica y Tecnología de Alimentos (IATA), Consejo Superior de Investigaciones Científicas (CSIC), Valencia, Spain; ^2^Departamento de Bioquímica y Biología Molecular, Universitat de València, Valencia, Spain

**Keywords:** iron deficiency, iron excess, iron homeostasis, iron metabolism, yeast, *Saccharomyces cerevisiae*, transcriptional regulation, post-transcriptional regulation

## Abstract

Iron is an essential micronutrient for all eukaryotic organisms because it participates as a redox cofactor in many cellular processes. However, excess iron can damage cells since it promotes the generation of reactive oxygen species. The budding yeast *Saccharomyces cerevisiae* has been used as a model organism to study the adaptation of eukaryotic cells to changes in iron availability. Upon iron deficiency, yeast utilizes two transcription factors, Aft1 and Aft2, to activate the expression of a set of genes known as the iron regulon, which are implicated in iron uptake, recycling and mobilization. Moreover, Aft1 and Aft2 activate the expression of Cth2, an mRNA-binding protein that limits the expression of genes encoding for iron-containing proteins or that participate in iron-using processes. Cth2 contributes to prioritize iron utilization in particular pathways over other highly iron-consuming and non-essential processes including mitochondrial respiration. Recent studies have revealed that iron deficiency also alters many other metabolic routes including amino acid and lipid synthesis, the mitochondrial retrograde response, transcription, translation and deoxyribonucleotide synthesis; and activates the DNA damage and general stress responses. At high iron levels, the yeast Yap5, Msn2, and Msn4 transcription factors activate the expression of a vacuolar iron importer called Ccc1, which is the most important high-iron protecting factor devoted to detoxify excess cytosolic iron that is stored into the vacuole for its mobilization upon scarcity. The complete sequencing and annotation of many yeast genomes is starting to unveil the diversity and evolution of the iron homeostasis network in this species.

## Introduction

Iron is a vital micronutrient for all eukaryotic organisms. Its redox activity and its ability to bind to multiple ligands enables iron to participate as a cofactor in the form of heme, iron-sulfur clusters (ISC), mononuclear iron or oxo-diiron centers in numerous biological processes including respiration, DNA replication and repair, ribosome biogenesis, translation, photosynthesis, biosynthesis of lipids and oxygen transport. Iron is one of the most abundant elements in the Earth’s crust, but the extremely low solubility of ferric iron (Fe^3+^) at physiological pH has dramatically restricted its availability to living organisms. Consequently, iron deficiency anemia has become the most common nutritional disorder worldwide, affecting two billion people, particularly women, children and older adults [reviewed in Chaparro and Suchdev (2019); Means (2020)]. The same redox properties that make iron indispensable for life also lead to cytotoxicity when present at high concentrations since it participates in Fenton reactions producing hydroxyl radicals that damage cells at the DNA, lipid and protein levels. Therefore, intracellular iron levels have to be tightly controlled. Defects in human iron homeostasis are directly related to disorders such as Friedreich’s ataxia, myopathies and encephalomyopathies, hemochromatosis, multiple mitochondrial dysfunction syndromes, and to increased risk of infections and cancer [reviewed in Stehling et al. (2014); Muckenthaler et al. (2017)]. Iron deficiency is also a concern in agriculture because it induces chlorosis and reduces photosynthesis, affecting both the yield and the nutritional value of crops [reviewed in Puig et al. (2007); Zhang et al. (2019)]. For all these reasons, studying the molecular mechanisms that regulate iron metabolism is important to understand iron-related physiological alterations, to deal with its nutritional defect consequences, and to develop medical treatments and agricultural applications. The budding yeast *S. cerevisiae* has been used as a model organism to study many aspects of iron homeostasis and regulation in eukaryotes. In fact, recent systems biology approaches have proposed a comprehensive mechanistic model for iron metabolism that has also been integrated into a whole-yeast metabolic model (Dikicioglu and Oliver, 2019; Lindahl, 2019). Here, we briefly review the mechanisms that control the adaptation of *S. cerevisiae* to changes in iron bioavailability.

## Regulation in Response to Iron Deficiency

### Transcriptional Activation of the Iron Regulon by Aft1 and Aft2

**FIGURE 1:**
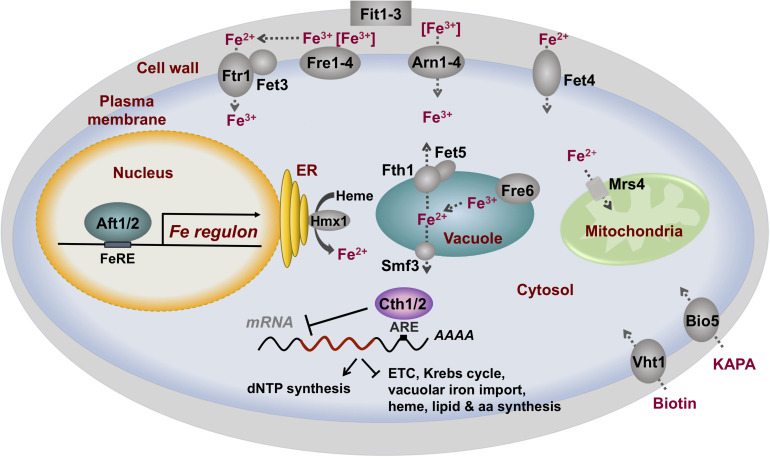
The transcriptional factors Aft1 and Aft2 activate the iron regulon in response to iron deficiency in *S. cerevisiae*. Iron regulon members include genes that encode for proteins that enhance the acquisition of extracellular iron, the mobilization and recycling of intracellular iron, iron-independent alternatives to iron-using processes, and a metabolic remodeling response of iron-dependent processes mediated by the mRNA-binding proteins Cth1 and Cth2, among other processes. KAPA: 7-keto-8-aminopelargonic acid.

In response to iron limitation, the yeast Aft1 and Aft2 (Aft1/Aft2) transcription factors accumulate in the nucleus, bind to iron-regulatory promoter elements (FeREs) with the consensus sequence PyPuCACCCPu (where Py is a pyrimidine and Pu is a purine), and activate the transcription of a group of genes collectively known as the iron regulon [reviewed by Philpott and Protchenko (2008); Kaplan and Kaplan (2009); Martins et al. (2018a)]. The following proteins belong to the yeast iron regulon: (1) the reductive iron uptake system, which is formed by four heme-containing cell surface metalloreductases (Fre1–Fre4), the copper-dependent high-affinity iron importer complex Fet3/Ftr1 and its copper-delivery proteins Atx1 and Ccc2, and the oxygen-independent low-affinity plasma membrane iron and copper transporter Fet4; (2) the non-reductive iron import machinery, which includes three cell wall mannoproteins (Fit1–Fit3) and four iron-xenosiderophore-specific transporters (Arn1–Arn4); (3) the vacuolar mobilization machinery constituted by the Fre6 metalloreductase, and the Fet5/Fth1 (paralogs of Fet3/Ftr1) and Smf3 iron exporters; (4) iron recycling proteins such as the Hmx1 heme oxygenase; (5) mitochondrial iron importers including Mrs4; (6) iron-independent alternatives to iron-using processes such as the biotin and 7-keto-8-aminopelargonic acid (KAPA) importers Vht1 and Bio5, respectively; and (7) the mRNA-binding proteins Cth1 and Cth2 implicated in iron metabolism remodeling (Figure 1). Recent studies have identified novel genes activated by Aft1 in response to iron depletion: (1) the trans-Golgi network K^+^/H^+^-exchanger gene *KHA1*, which product facilitates copper loading into apo-Fet3 multicopper-ferroxidase (Wu et al., 2016); (2) *MMT1* and *MMT2* mitochondrial iron exporter genes (Li et al., 2020); (3) *RNR1*, encoding for the catalytic subunit of the iron-dependent ribonucleotide reductase (RNR) enzyme; and (4) *RNR1* transcriptional activator *IXR1* (Ros-Carrero et al., 2020).

Genetic and biochemical studies have demonstrated that ISC assembly and export machineries are directly involved in the post-transcriptional regulation of iron metabolism in higher eukaryotes (through the iron regulatory proteins, IRPs) and transcriptional regulation and sensing of iron homeostasis in yeast cells via specific transcription factors including Aft1, Aft2, and Yap5 (see below) [reviewed in Muhlenhoff et al. (2015); Martinez-Pastor et al. (2017); Gupta and Outten (2020)]. Upon iron-sufficient or high-iron conditions, the activity of the Aft1/Aft2 transcription factors is limited to avoid the harmful consequences of excess iron acquisition. Curiously, yeast Aft1/Aft2 do not respond to cytoplasmic iron levels; instead they sense a mitochondrial signal from ISC biosynthesis that inhibits their function in iron-replete conditions (Chen et al., 2004; Rutherford et al., 2005; Hausmann et al., 2008). Thus, yeast cells with an impaired ISC system induce the extracellular iron uptake systems and accumulate iron in mitochondria (Kispal et al., 1999; Chen et al., 2004; Rutherford et al., 2005; Hausmann et al., 2008). Under iron-sufficient conditions, an uncharacterized sulfur-containing compound, denoted X-S, is exported to the cytoplasm by the yeast Atm1 (human ABCB7) mitochondrial transporter. After multiple steps, the X-S molecule is converted into a [2Fe–2S]^2+^ cluster that is coordinated via cysteine residues to a homodimer of monothiol glutaredoxins (Grx3 or Grx4) and two glutathione molecules. Finally, the Bol2/Fra2 protein transfers the ISC to the Aft1/Aft2 transcription factors, which homodimerize, decreasing their affinity for DNA and being exported to the cytosol (Li and Outten, 2012, 2019; Poor et al., 2014). A recent study has shown that the mitogen-activated protein kinase (MAPK) Hog1 limits Aft1 function by directly phosphorylating specific serine residues and promoting its nuclear export (Martins et al., 2018b). Further data have demonstrated that when subcellular localization is impaired, Aft1/Aft2 transcriptional activity is still regulated via iron-dependent DNA-binding (Ueta et al., 2012; Poor et al., 2014). Nowadays, iron-sensing is an area of intense research in both yeast and mammals.

### Post-transcriptional Remodeling of Iron Metabolism by the mRNA-Binding Protein CTH2

In response to iron limitation, yeast cells activate the expression of two tristetraprolin family members (Wells et al., 2017), called Cth1 and Cth2, characterized by the presence of a conserved Cx_8_Cx_5_Cx_3_Hx_18_Cx_8_Cx_5_Cx_3_H tandem zinc finger (TZF) motif that directly interacts with adenosine and uridine-rich elements (AREs) in the 3′ untranslated region (3′UTR) of target mRNAs to post-transcriptionally limit their expression [(Puig et al., 2005, 2008; Pedro-Segura et al., 2008; Prouteau et al., 2008); reviewed in Martinez-Pastor et al. (2013a)]. *CTH1* expression remains low, whereas *CTH2* is highly induced upon iron deficiency (Puig et al., 2005, 2008). The higher relevance of Cth2 in the adaptation of yeast cells to iron depletion is highlighted by the growth defect that *cth2*Δ mutant cells display under these conditions, which is exacerbated in *cth1*Δ*cth2*Δ double mutants (Puig et al., 2005). Cth2 is a highly unstable nucleocytoplasmic shuttling protein (Vergara et al., 2011; Romero et al., 2018b). A nuclear localization signal (NLS) embedded in its TZF motif enables its import to the nucleus, where it co-transcriptionally associates to ARE-containing mRNAs (Prouteau et al., 2008; Vergara et al., 2011). Then, Cth2 either promotes the nuclear degradation of its bound transcripts or their export to the cytosol where it facilitates their 5′ to 3′ ARE-mediated decay (AMD) and translational inhibition (Pedro-Segura et al., 2008; Prouteau et al., 2008; Vergara et al., 2011; Ramos-Alonso et al., 2018a). Cth2 amino-terminal region is important for both AMD and translation repression, whereas its carboxy-terminal domain is only required to inhibit protein synthesis (Ramos-Alonso et al., 2018a, 2019). Cth2 does not contain any nuclear export signal; instead it relies on the mRNA export machinery to exit the nucleus (Vergara et al., 2011). Importantly, Cth2 nucleocytoplasmic shuttling is absolutely necessary for its mRNA regulatory function (Vergara et al., 2011). Very recent protein interaction data have demonstrated that Cth2 enters the nucleus in association to the Dhh1 RNA helicase and the Pop2/Caf1 deadenylase proteins, whereas the 5′ to 3′ Xrn1 exonuclease is only recruited after Cth2-binding to its target mRNAs (Perea-Garcia et al., 2020). Thus, when mRNA decay is impaired, Cth2 protein is trapped in microscopically visible foci where mRNA turnover takes place (cytosolic processing bodies), probably because the Cth2 TZF-embedded NLS is not available (Pedro-Segura et al., 2008). Yeast Cth2 is an excellent model to study how eukaryotic mRNA-binding proteins post-transcriptionally control mRNA expression.

In response to iron-deficient conditions, Cth2 post-transcriptionally inhibits the expression of ARE-containing mRNAs that encode proteins that directly bind iron or that are involved in iron-consuming pathways [reviewed in Sanvisens and Puig (2011); Philpott et al. (2012); Outten and Albetel (2013)]. The most numerous set of Cth2 mRNA targets encode for components of the mitochondrial electron transport chain, a highly iron-consuming process that is not essential for yeast cells, since they mostly ferment even in aerobic conditions. Consistent with this, Cth2 expression limits oxygen consumption (Ramos-Alonso et al., 2018b; Sato et al., 2018). Genome-wide studies have shown that, in addition to regulating respiration, Cth2 targeted mRNAs are implicated in other iron-dependent pathways including components of the tricarboxylic acid (TCA) cycle (such as aconitase and succinate dehydrogenase), the biosynthesis of unsaturated fatty acids, ergosterol and sphingolipids, and the synthesis of numerous amino acids (e.g., leucine, lysine, methionine, or glutamate) and cofactors such as biotin and lipoic acid (Puig et al., 2005, 2008). Cth2 also limits the accumulation of iron into the vacuole when iron levels are low by promoting the degradation of the *CCC1* transcript encoding for the vacuolar iron importer (Puig et al., 2005). The overexpression of a functional *CTH1* or *CTH2* gene is highly cytotoxic (Thompson et al., 1996; Pedro-Segura et al., 2008; Romero et al., 2018b), so yeast cells have to tightly control their expression. Interestingly, both mRNAs contain AREs that allow a negative cross- and auto-regulation that limits their expression and that is important for the activation of respiration and for optimal adaptation to the transit from iron-deficient to iron-sufficient conditions (Martinez-Pastor et al., 2013b). Remarkably, Cth2 does not only down-regulate iron-consuming processes, but it also preferentially promotes iron-dependent activities that are essential or highly important for cells. An illustrating example is RNR, an oxo-diiron-containing enzyme that catalyzes the *de novo* synthesis of deoxyribonucleotides (dNTPs). Under normal conditions, the activity of RNR is limited by the different subcellular localization of its cytosolic large catalytic subunit R1 and its nuclear iron-containing small subunit R2. In response to iron deprivation, Cth2 promotes the degradation of the ARE-containing *WTM1* mRNA, which encodes for a nuclear R2 anchoring protein (Sanvisens et al., 2011). In this way, Cth2 facilitates the export of the small R2 subunit to the cytosol and the assembly of a functional RNR holoenzyme (Sanvisens et al., 2011). Therefore, Cth2 is a central coordinator of iron metabolism that prioritizes iron utilization in essential processes over dispensable iron-using ones.

### Regulation of Metabolic and Cellular Processes in Response to Iron Depletion

**FIGURE 2:**
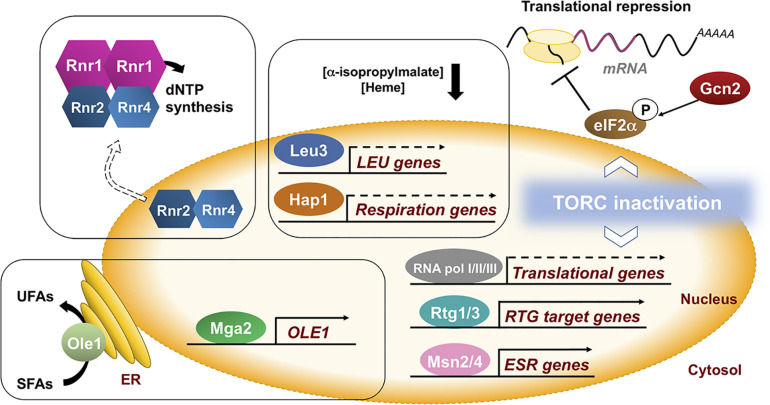
Yeast adaptation to iron deficiency requires the remodeling of many cellular processes. In *S. cerevisiae*, multiple iron-related processes indirectly respond to iron deficiency. The lack of iron leads to a decrease in the availability of iron-dependent metabolites including amino acid intermediates, heme, unsaturated fatty acids (UFAs) and deoxyribonucleotides (dNTPs), leading to changes in the expression of genes implicated in their biosynthesis. Moreover, global nutrient signaling pathways (TORC1) and environmental stress responses (ESR) respond to iron limitation by causing a bulk decrease in transcription and translation, and the activation of specific metabolic pathways such as the mitochondrial retrograde (RTG) response. These observations highlight the huge amount of direct and indirect connections between iron and cellular metabolism. Further detailed studies are necessary to fully decipher how eukaryotic cells sense iron starvation and transduce this signal to a wide range of cellular processes.

In response to iron deficiency, the decrease in activity of some iron-dependent enzymes limits the levels of key metabolic intermediates that are used as coactivators of specific transcription factors (Ihrig et al., 2010). For instance, in response to low iron, there is a decrease in the expression of genes from the iron-dependent branched-chain amino acid biosynthesis pathway due to a drop in the levels of the metabolic intermediate α-isopropylmalate, which serves as a coactivator of the Leu3 transcription factor (Ihrig et al., 2010; Figure 2). Similarly, the decrease in heme levels that occurs under iron starvation conditions limits the activity of the Hap1 transcription factor, a regulatory process that contributes to the down-regulation of mitochondrial respiration (Ihrig et al., 2010; Figure 2). Furthermore, multiple molecular markers indicate that the nutrient-signaling pathway that depends on the target of rapamycin complex 1 (TORC1) is also inhibited during the advance of iron deficiency, although the triggering signal is currently unknown and would require further studies (Romero et al., 2019; Figure 2). As a consequence, there is a decrease in the transcriptional activity of all RNA polymerases that causes the down-regulation of genes encoding for ribosomal proteins (RPs) and ribosome biogenesis (RiBi) factors, as well as a significant drop of rRNAs and tRNAs levels that finally impair global translation (Romero et al., 2019, 2020; Ros-Carrero et al., 2020; Figure 2). Interestingly, iron deficiency also promotes the Gcn2-dependent phosphorylation of the translation initiation factor eIF2α, probably due to the presence of uncharged tRNAs, contributing to the repression of bulk translation (Romero et al., 2020; Figure 2). Consistent with a defect at the initiation step, the translation of the *GCN4* mRNA, which depends on short upstream open reading frames (uORFs), is enhanced (Romero et al., 2020). It is important to stress that translation is a highly energy consuming process that requires the conserved ISC-containing Rli1 (human ABCE1) protein for ribosome biogenesis and recycling (Kispal et al., 2005; Yarunin et al., 2005; Young et al., 2015).

Iron deficiency also triggers the activation of various signaling pathways beyond the Aft1/Aft2 iron regulon (Martins et al., 2018a). Upon iron scarcity, there is an increase in the transcription of genes belonging to the mitochondrial retrograde (RTG) response (*CIT1*, coding for the mitochondrial isoform of citrate synthase; *ACO1*, aconitase, and *IDH1* and *IDH2*, encoding NAD^+^-dependent isocitrate dehydrogenases) via the Rtg1-Rtg3 transcription factor complex (Figure 2), probably due to TORC1 inhibition or to mitochondrial dysfunction (Romero et al., 2019). The activation of the RTG response during iron deficiency may provide sufficient α-ketoglutarate as a nitrogen donor for biosynthetic processes. Moreover, a decrease in the activity of the iron-dependent Δ9 fatty acid desaturase Ole1 in iron-deficient conditions causes a drop in the levels of unsaturated fatty acids (UFAs) that releases the endoplasmic reticulum (ER)-anchored Mga2 transcription factor and activates the transcription of *OLE1* (Romero et al., 2018a; Figure 2). UFA regulation of Mga2 is essential for growth in iron-depleted conditions (Romero et al., 2018a; Jorda et al., 2020). In fact, the levels of UFAs have been reported to be crucial for the Aft1-dependent activation of the iron regulon, although no mechanistic clues have been provided yet (Jorda et al., 2020). Moreover, both iron deficiency and defects in the mitochondrial ISC biogenesis activate the Mec1-Rad53-Dun1 DNA damage checkpoint kinase cascade that, in combination with Cth2 and Aft1 regulatory factors (see above), promote the activity of the iron-dependent RNR enzyme at multiple levels [(Sanvisens et al., 2011, 2014, 2016; Pijuan et al., 2015; Ros-Carrero et al., 2020); reviewed in Sanvisens et al. (2013); Puig et al. (2017)]. Upon iron limitation, Dun1 kinase enhances dNTP synthesis by promoting the degradation of the R1 inhibitor protein Sml1, and the R2 import protein Dif1 (Sanvisens et al., 2014, 2016). Future studies would determine whether the DNA damage checkpoint kinase cascade is activated when iron is scarce as a consequence of defects in the activity of the iron-dependent DNA polymerases and DNA repair enzymes (Puig et al., 2017).

Iron starvation also initiates the environmental stress response (ESR) program (Romero et al., 2019), that includes the transcriptional activation, via the general stress transcription factors Msn2 and Msn4, of ∼300 mRNAs mostly implicated in protecting cells against adverse stress conditions, and the repression of ∼600 transcripts encoding RPs, RiBis and other factors implicated in translation (Gasch et al., 2000). The substitution of iron-using processes by iron-independent alternatives is also a broad yeast response. In addition to the replacement of the iron-dependent biosynthesis of biotin by its uptake, yeast cells also decrease the expression of the ISC-dependent glutamate synthase enzyme Glt1 in favor of a glutamate dehydrogenase iron-independent route for nitrogen assimilation (Belli et al., 2004; Shakoury-Elizeh et al., 2004).

## Regulation in Response to Iron Excess

The yeast iron detoxification response is mostly triggered by the transcriptional activator protein of the Yap family, Yap5 [reviewed in Li and Ward (2018); Rodrigues-Pousada et al. (2019)]. Yap5 associates to Yap response elements (YREs) within the promoter of its target genes independently of iron levels, but only activates transcription when it associates to two [2Fe–2S] clusters through conserved cysteine-rich domains in a mitochondrial ISC-dependent but Grx3/4-independent manner (Li et al., 2008, 2012; Rietzschel et al., 2015). Thus, in response to high-iron, Yap5 activates the transcription of: (1) the *CCC1* vacuolar importer gene, which is the main iron storage yeast facilitator; (2) the *GRX4* monothiol glutaredoxin, which binds and transfers the iron-derived mitochondrial signal to Aft1 and Aft2 transcription factors limiting their activity; (3) *TYW1*, which encodes for a cytosolic [4Fe–4S] cluster-containing enzyme probably implicated in iron buffering; and (4) the copper metallothionein gene *CUP1* to protect cells from oxidative stress (Li et al., 2011; Pimentel et al., 2012).

Curiously, cells lacking *YAP5* are less sensitive to iron than *ccc1*Δ cells, suggesting that other transcriptional factors contribute to the expression of *CCC1* (Pimentel et al., 2012). In this sense, a recent study has discovered that yeast cells lacking the low-glucose sensor Snf1 or other components of this kinase complex display defects in *CCC1* expression under high-iron conditions that lead to iron sensitivity (Li et al., 2017). Remarkably, the Snf1 activation of *CCC1* does not depend on Yap5 or ISC biogenesis, but utilizes Msn2 and Msn4 transcription factors and other unidentified regulatory factors (Li et al., 2017). Consistent with this, the *msn2*Δ*msn4*Δ double mutant is sensitive to iron and the Msn4 protein translocates to the nucleus in response to excess iron (Du et al., 2012; Li et al., 2017). Further studies are necessary to decipher how iron modulates the activity of the Snf1 kinase complex since no changes in Snf1 phosphorylation have been reported upon exposure to high iron.

## Iron Homeostasis Diversity in *S. cerevisiae* Yeast Strains

The utilization over the past decades of laboratory *S. cerevisiae* strains has allowed the characterization of the main features that govern eukaryotic iron homeostasis, including iron sensing, regulation, acquisition, distribution, storage, and utilization. However, the recent sequencing of the genome of numerous natural and human-domesticated yeast strains is opening novel horizons for studying iron homeostasis diversity and evolution that should be pursued in the future (Peter et al., 2018). In addition to the reductive strategy for iron uptake, some budding yeasts are also able to synthesize and acquire iron via siderophores. A recent phylogenomic study has suggested that the genes implicated in the biosynthesis and utilization of the iron-binding molecule pulcherrimin were ancestral to *S. cerevisiae*, although lost in the majority but not all lineages (Krause et al., 2018). Another report has uncovered the acquisition by a group of budding yeasts of a whole bacterial operon implicated in siderophore iron transport through horizontal transfer (Kominek et al., 2019). A genetic study has unveiled the diversity of wild Malaysian *S. cerevisiae* strains, which have adapted their iron homeostasis network to their natural environment (Lee et al., 2013). These strains are particularly sensitive to iron due to defects in *YAP5* gene and especially in their *CCC1* vacuolar iron transporter allele, but display an *AFT1* allele that improves their adaptation to iron-deficient conditions (Lee et al., 2013). Whole-genome analyses of *S. cerevisiae* strains used for the production of sherry-like wine have also revealed an improved iron uptake system and an increased sensitivity to iron probably due to the presence of *AFT1*, *FRE* and *FIT* alleles also found in flor yeasts (Eldarov et al., 2018). In a broad phenotypic characterization of *S. cerevisiae* strains from different geographical and source origins, yeast strains were classified as iron-resistant and iron-sensitive (Martinez-Garay et al., 2016). Curiously, the most iron-resistant strains accumulate less iron than the sensitive ones, and grow poorly in iron-deficient conditions, whereas the iron-sensitive strains have better adapted to low iron environments (Martinez-Garay et al., 2016). A more detailed study of both the genotype and phenotype of these and other yeast strains will definitely contribute to a better understanding of iron homeostasis and evolution.

## Author Contributions

LR-A and SP wrote the original draft. MM-P and AR reviewed and edited the manuscript. All authors contributed to the article and approved the submitted version.

## Conflict of Interest

The authors declare that the research was conducted in the absence of any commercial or financial relationships that could be construed as a potential conflict of interest.
